# Survivors’ Solidarity and Attachment in the Immediate Aftermath of the Typhoon Haiyan (Philippines)

**DOI:** 10.1371/currents.dis.2fbd11bd4c97d74fd07882a6d50eabf2

**Published:** 2017-01-09

**Authors:** Andrea Bartolucci, Michele Magni

**Affiliations:** Engineering and the Built Environment, Anglia Ruskin University, Chelmsford, Essex, United Kingdom; Department of Life and Environmental Sciences, Università Politecnica delle Marche, Ancona, Italy

## Abstract

Introduction: Anti-social behavior and self-preservation are often assumed to be normal responses to threats and disasters; on the contrary, decades of research and empirical studies in social sciences showed that pro-social behaviors are frequently common and that solidarity is the typical response to a variety of threats. The main objective of this study is to investigate and describe survivors’ behavior, especially solidarity, according to the presence of familiar persons and to the perception of physical danger, elaborating the framework of Mawson’s social attachment theory.

Methods: In order to investigate these relationships, a behavioral research was carried out involving 288 people affected by the December 8th 2013 Haiyan Typhoon (Yolanda).

Results: Results revealed that solidarity was predominant and people reacted collectively and actively taking part in relief activities. Furthermore, we found strong solidarity and help towards strangers and unfamiliar persons.

Discussion: Investigating how people react is essential to develop a more efficient and effective response strategy, especially in the immediate aftermath of a disaster when disaster managers have little control of the situation and people rely on themselves; the natural tendency to help others can be essential to reduce losses and to fill the temporal gap between the event and the arrival of the organized relief unit.

## Introduction

Many myths and incorrect beliefs are common about disasters; anti-social and irrational behaviors are usually assumed to be the most common response to danger and disasters^1^^,^ 2 ^,^ 3 ^,^ 4 ^,^ 5 panic, for example, is frequently and erroneously used to describe disaster response^6^^,^ 7, despite it is a rare phenomenon that occurs only in presence of specific affective/cognitive factors^8^. Decades of research and empirical assessment on disasters and emergencies revealed that what really happens after a disaster frequently differs from what the conventional wisdom would suggest: victims tend to respond effectively and creatively^9^ and the behavior in such circumstances is very meaningful and far from many conceptions of irrationality^7^^,^ 8. Uncertainty and confusion characterize the disaster scenario but the fear felt during the early phase of the emergency, instead of leading to maladaptive behavior, turns in altruistic acts with people start looking for ways to secure their own and others safety^10^. Survivors’ decision to behave, adapt and respond in the immediate aftermath of a disaster is anticipated by an extended period when people mill about^11^ and starts with the assessment of a changed social and physic environment^12^. Within this process, the individuals’ assessment of personal risk (or the perception of) as variables of environment perception is one of the main factor influencing the decision 12 ^,^ 13 ^,^ 14. More, due to the fact that people are used to move in group^12^^,^ 13 ^,^ 14 ^,^ 15 ^,^ 16, survivors’ behavior cannot be merely explained as sum of behaviors of individuals who influence each other. Survivors can be with familiar people, strangers and a combination of both. In any case, people use to maintain groups, by seeking the proximity of familiar people, or to create a new relationship with strangers who share the same feeling and fate. Shared identity, in fact, resulting from the sharing of the same emergency experience, enhances the expression of solidarity^16^, mutual aid and sociality^8^^,^ 17 ^,^ 18 ^,^ 19 ^,^ 20. There are many studies on people helping familiars persons in different emergencies and disaster situations^9^^,^ 10 ^,^ 12 ^,^ 16; in 2005, Mawson proposed the “social attachment model” according to which seeking the proximity of familiar persons and places rather than fleeing is the typical response to a variety of threats^20^. This theory describes the response to threats and disasters as a movement towards a situation that is perceived as safe (but not necessarily objectively safe). According to Mawson’s discussion, combining the factors of perceived physical danger (called precipitating condition) and location of attachment figures (called predisposing condition) results in a four typology behavioral model that encompasses a wide spectrum of collective responses to threat and disasters (Fig. 1).

**Mawson's profiles:**
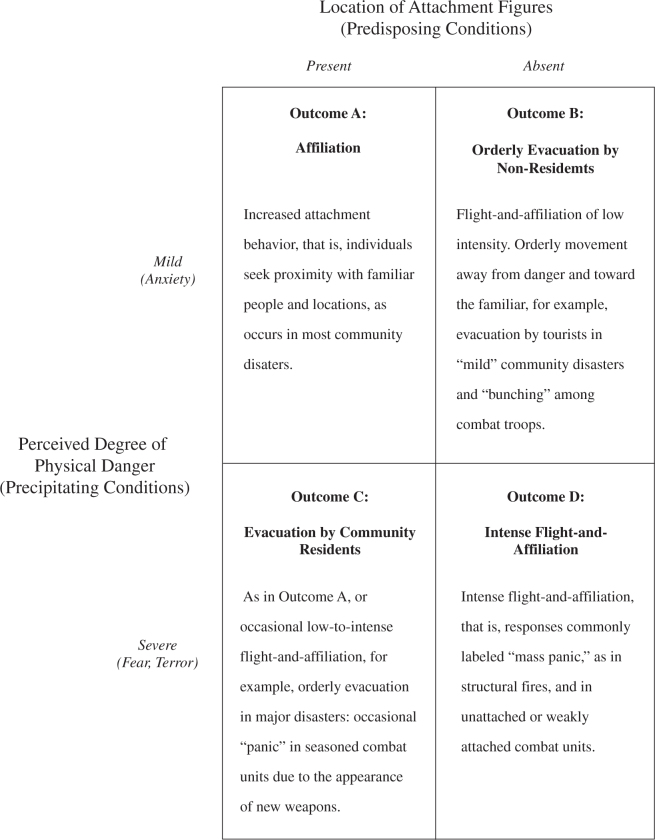
Four-fold typology of individual and collective reactions to threat and disaster as proposed by Mawson.

This paper was criticized by some authors, especially regarding the undefined nature of the attachment behavior^21^ and the lack of empirical research and new contributions to the current theory of social behavior in disaster^22^. Moreover, this theory doesn’t describe and explain the bonds that rise during a disaster among people that are not familiar or known. Although the assumptions of the social attachment model have been strongly criticized, the framework of Mawson’s model should be held useful to describe ideal responses patterns or collective actions. In this paper we investigate and analyzed survivors’ behavior in the immediate aftermath of a disaster; we focus on solidarity and mutual help and we studied how social variables, such as the presence of familiar people or strangers, and environmental aspects, such as the perception of danger influence people’s response. The main aim is to understand and describe the actual behavior of survivors in emergency and disasters in order to help disaster planners and managers to develop better response strategies.

## Methods

In this paper we designed a case study survey^23^ with the purpose of investigating the behavior of victims in the aftermath of a real event that was carried out after the Haiyan (Yolanda) typhoon. The typhoon (category 5) hit and devastated portions of Southeastern Asia, more specifically several regions in the Eastern cost of the Philippines. According to the National Disaster Risk Reduction and Management Council^24^ of the Republic of Philippines, the event killed at least 6,300 and caused 28.689 injured people and 1601 missing persons. The relief team operated with many difficulties and this forced people to react in the immediate aftermath without a specific coordination and support of relief teams. The study area is San Roque (Fig. 2), a seaside barangay (municipality) in Tanauan (Leyte), one of the most affected communes of the Eastern Visayas Region (VIII), where Municipal Local Government reported 1,200 people dead and almost 2,000 missing, out of a population of almost 50,000.

**Study area:**
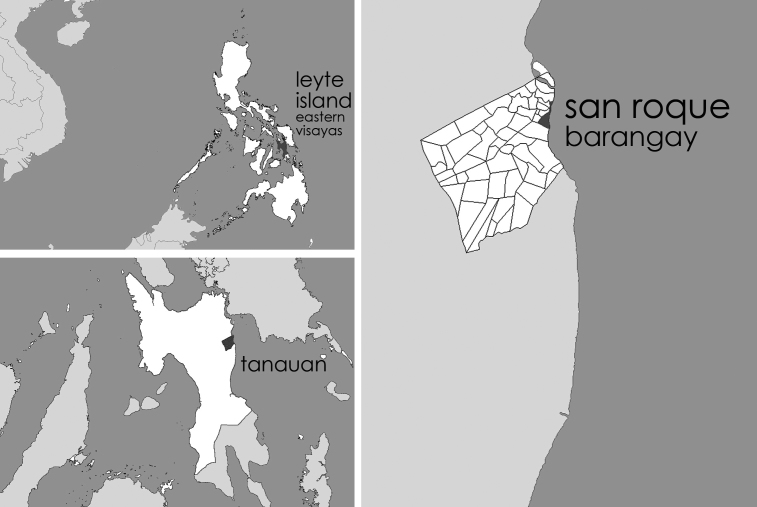
Location of the study area: Barangay San Roque in Tanauan, Leyte, Eastern Visayas Region (VIII)

**Research design**

A cross-sectional survey was conducted because it is able to provide highly viable and excellent data on behavioral attitude of people after disasters^25^. Between December 11th and December 16th 2013, 288 volunteers, who experienced the Typhoon Haiyan in first person, were asked to fill a structured questionnaire. Given the chaotic and challenging scene which prevails in the aftermath of disasters and the difficulty to trace a demographic profile of the population because the lack of available data, the sample was selected according to people’s ready availability to be recruited^26^. Considering that the questionnaire delivery mode also has to be tailored on the so called “survey-taking climate” (e.g. social context in which the research is being undertaken), it was opted for the adoption of a mixed method which integrated face-to face contact with self-completion of the questionnaire^27^. The contact with people who experienced the event occurred in two moments: a public meeting in the meeting hall of the city and during the distribution of the relief supplies in the town hall. The participants were supported by investigators in order to provide an in-depth insight into the purposes of this research and to support them in the completion of the questionnaire in an anonymous way, reducing the possibility to obtain a low return rate due to misunderstandings or social desirability bias. The questionnaires were administered in Tagalog, an Austronesian language spoken as first language by a quarter of the population in the Philippines and as second language by the majority of people^28^. An alternative English version was available. Data collection and analysis were carried out following the policies adopted by the Università Politecnica delle Marche (UNIVPM). Each responder provided verbal informed consent prior to participation and voluntarily decided whether to participate or not. The authors collected no identifying participant data. All the interviewees were informed about the scientific purpose of the study survey and on the use of data collected.

**Materials**

Starting from the four-fold typology of responses proposed by Mawson (Fig. 1), a structured questionnaire was developed in order to test the spectrum of collective responses to threats in presence of “predisposing conditions” (shortened with PRED), that describe the proximity of attachment figures, and “precipitating conditions” (shortened with PREC), that quantify the perception of the physical danger (and not the degree of perceived danger used by Mawson). The attachment figures were defined for participant as familiars, relatives, friends or even known people. A structured format containing dichotomous questions was chosen in order to obtain answers as much objective as possible; except for the question related to PRED and PREC, the questionnaire contained only polar questions. After a set of questions, aimed at evaluating the demographic profile, respondents were asked to express their perception of danger (mild/severe) and whether they were in presence or not (present/absent) of attachment figures. Furthermore, they answered to a set of questions aimed at investigating their behavior in the aftermath of the Typhoon. More specifically, respondents indicated if they decided to leave instead of stay and wait for the relief, if they relied upon a person who appeared to be a leader, if they interact with strangers and unknown people, if they engaged in a response behavior when dealing with the situation, if they took part in rescue activities, and finally if they tried to collect additional information on the event. The questions were not directly correlated one to each other, but they intentionally followed a sequence that aimed at addressing the decision-making process over the time. The relationship between PRED and PREC conditions and behavioral questions was explored by performing Chi-square tests for independence with Bonferroni correction for multiple comparisons. According to Mawson’s four-fold typology response, the sample was divided in behavioral profiles and the association between each profile and behavioral questions was investigated by applying Chi-square tests for independence with Bonferroni correction for multiple comparisons. Eventually, results were combined into an alternative profile chart that describes victims’ behavior in response to threat and disaster.

## Results

Respondents were averagely 37.3 years old (SD ± 16.1) with 10 years of median education level. The majority of them were females (62%). Ninety-one per cent of the respondents already experienced an extreme weather event. There is a fine balance between respondents who were with attachment figures (55%) in the aftermath of the event and those who were alone or with strangers (45%). More than three out of four of them perceived a severe level of physical danger (76%). Overall, most of the respondents (82%) tended to stay on the site instead of leave it with a narrow majority of them (51%) engaged in an information seeking behavior. Sixty-three percent of the respondents claimed to have relied upon a person that appeared as a leader and 69% of them reported to adopt a response behavior after the Typhoon. Despite the majority of interviewed people (59%) took part in rescue activities, less than half of them interacted with strangers (47%). The associations of PRED and PREC with each of the behavioral variables were analyzed either singularly or in the four-fold form, which derived from the combinations of the PRED (present/absent) and PREC (mild/severe) outcomes. Chi-square tests for the independence (Yates continuity correction) showed the same significant association only for the variable “having relied upon a leader” (PRED - χ^2^ (1) = 8.26, p<.005; PREC - χ^2^ (1) = 4.73 p<.05). Table 1 reports the cross-tabulation between each behavioral question and the two conditions (predisposing and precipitating). Bonferroni correction (α level = 0.008) was applied in order to control the familywise error rate associated with the multiple comparisons performed in the Chi-square test.

**Table 1: Behavior and variables:** Behavioral questions results according to predisposing and precipitating conditions

BEHAVIORS		PRESENCE OF ATTACHMENT FIGURES (PRED)			DEGREE OF PERCEIVED PHYSICAL DANGER (PREC)		
		Presence	Absence	χ^2 ^(df)	Presence	Absence	χ^2^ (df)
Decide to stay on site	Yes	78%	86%	2.34 (1)	65%	87%	15.71 (1) *
	No	22%	14%		35%	13%	
Collect information	Yes	39%	66%	19.62 (1) *	44%	54%	1.88 (1)
	No	61%	34%		56%	46%	
Rely upon a leader	Yes	70%	53%	8.26 (1) *	51%	66%	4.73 (1)
	No	30%	47%		49%	34%	
Engage in response behavior	Yes	79%	56%	16.45 (1) *	68%	69%	.08 (1)
	No	21%	44%		32%	31%	
Interact with strangers	Yes	51%	42%	1.95 (1)	30%	53%	9.39 (1) *
	No	49%	58%		70%	47%	
Participate in rescue	Yes	68%	49%	10.16 (1) *	29%	69%	2.34 (1)
	No	32%	51%		71%	31%	

*significant at 0.008 level (Bonferroni correction)

As evincible by results, people were more likely to rely upon a leader in presence of attachment figures while perceiving severe physical danger. Considering the PRED, people were more likely to adopt an information seeking behavior in absence of attachment figures (χ^2^ (1) = 19.62, p<.001), while they were more likely to engage in response behavior (χ^2^ (1) = 16.45, p<.001) and to participate in rescue activities (χ^2^ (1) = 10.16, p<.005) in presence of attachment figures. With regard to the PREC, people were more likely to leave the scene when perceiving mild physical danger (χ^2^ (1) = 15.71, p<.001), and to interact with strangers when they perceived severe physical danger (χ^2^ (1) = 9.39, p<.005). Once cross-tabulated with the behavioral variables and combined to obtain the four-fold response typology, PREC and PRED showed the results reported in Table 1. A further set of Chi-square tests for independence had been performed to evaluate the associations between the attitude of the respondents towards each of the behavioral variables and the response profile they belong to. Results of the analysis are shown in Table 2.

**Table 2: Behavior and Profiles:** Behaviors of the four different profiles

	Profiles					
Behaviors		A	B	C	D	χ^2^ (df)
Decide to stay on site	Yes	48%	91%	89%	85%	30.64 (3) *
	No	52%	9%	11%	15%	
Collect information	Yes	21%	75%	46%	63%	31.5 (3) *
	No	79%	25%	54%	37%	
Rely upon a leader	Yes	56%	46%	75%	55%	15.7 (3)*
	No	44%	54%	25%	45%	
Engage in active behavior	Yes	81%	49%	79%	58%	18.53 (3)*
	No	19%	51%	21%	42%	
Interact with stranger	Yes	36%	25%	47%	57%	13.34 (3)*
	No	64%	75%	53%	43%	
Participate in rescue	Yes	42%	9%	76%	59%	50.24 (3)*
	No	58%	91%	24%	41%	

*significant at 0.008 level (Bonferroni correction)

Statistically significant associations were found between four-fold profiles and the following behavioral questions: decision to stay on site in the aftermath of the event (χ^2^ (3) = 30.64, p<.001), with only Profile A exceeding the 50%; the information seeking behavior (χ^2^ (3) = 31.5, p<.001), with only Profiles B and D exceeding the 50%; the confidence in a leader (χ^2^ (3) = 15.7, p<.005), with only the Profile B below the 50% threshold; the engagement in an response behavior (χ^2^ (3) = 18.53, p<.001), with again only the Profile B under the 50% threshold; the interaction with strangers (χ^2^ (3) = 13.34, p<.01), with Profile D the unique over the 50%; and the participation in rescue activities (χ^2^ (3) = 50.24, p<.001), with Profiles A and B below the 50% threshold. The results of behavioral questions for each of the Profiles were plotted in a Kiviat diagram in order to provide a displayable and more intuitive version (Fig. 3).

**Kiviat diagrams:**
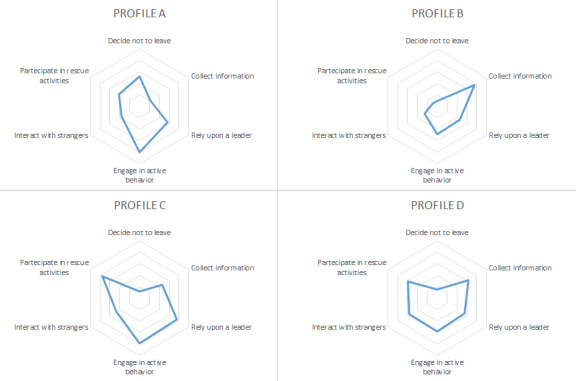
Kiviat diagrams shows behavioral responses for each profile

## Discussion

Survivors’ behavior in the aftermath of a disaster is mostly characterized by solidarity and collaboration within groups (pre-existing and emergent) instead of anti-social behavior and self-preservation. Such behaviors are influenced by many factors but the presence of attachment figures and the perception of danger play a pivotal role in decision-making process and reaction to emergency and disaster. Results showed that the most common response is to stay on site; only a part of the individuals in presence of attachment figures and with a perception of low physical danger left the scene after they have recreated the pre-existing group and when all the members were safe. The confidence on a person that drives the decisions of the rest of the group (leader) predominates in presence of attachment figures, in which pre-existing norms, roles and bonds keep working and driving the decision-making process of the whole group. However, the overall behavior was active and collaborative, especially for people in presence of attachment figures while the perception of danger seemed not to be a considerable driver. Solidarity and collaboration were strong and directed even to strangers and unknown people, expressing the same stressful feeling and trying to solve the situation together. The decision to take part in rescue activities was strongly influenced by the presence of familiar people; as mentioned above, in absence of familiar people and with perception of low level of physical danger, people were less collaborative and less likely to take part in rescue operations. The results of the analysis were combined in a new four-fold typology model that describes the individual and collective reactions focused on solidarity (Fig. 4).

**Study profiles:**
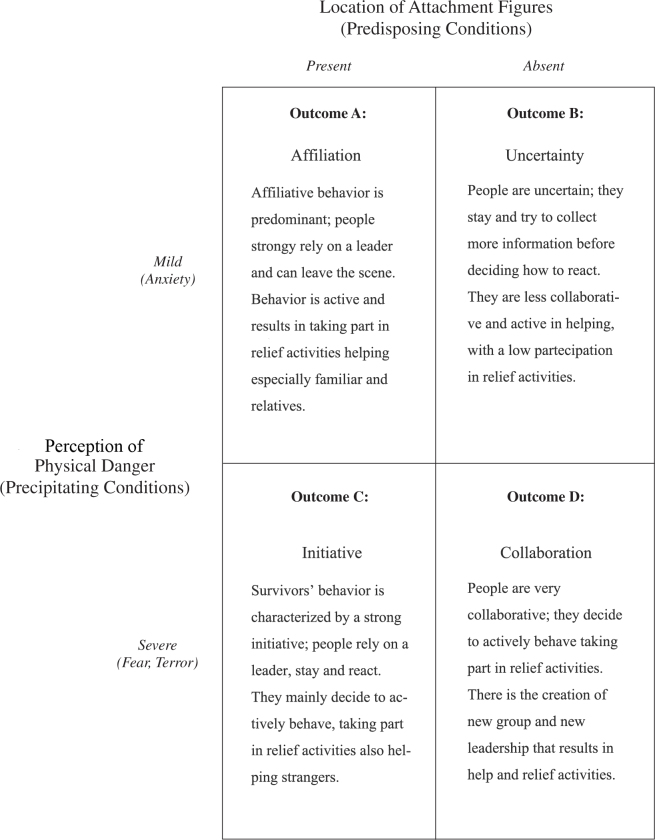
Alternative four-fold typology model of individual and collective reactions based on the results of this study

**Profile A**

Respondents who were in presence of attachment figures and with perception of mild physical danger display attachment behavior. Individuals seemed to seek and reinforced the pre-existing group by relying upon a leader and leaving the scene when the members were safe; more, they behaved actively taking part in relief activities but collaborated less with strangers.

**Profile B**

The perception of mild physical danger combined with the absence of attachment figures nearby, especially a leader, made the milling process and the reduction of uncertainty longer: this pushed people to collect further information. However, there were few possibilities to engage in response behavior, interacting with strangers and taking part in rescue activities.

**Profile C**

Respondents in presence of familiar persons and with a perception of high physical danger decided to stay in the aftermath and reacted very actively. Again, the figure of the leader assumed an important role. They strongly committed to in relief activities, even helping and collaborating with strangers and unknown people.

**Profile D**

People that perceived physical danger as high and with no familiar people in the proximity were collaborative and likely to interact with people around, even though they were strangers. Possibly, a strong interaction with the other victims was the result of an increasing sharing of feeling and common faith.

This study confirmed that people express strong solidarity, helping both attachment figures and also unknown people; it also underlined the affiliative and social nature of the spontaneous reaction. Nevertheless, survivors’ behaviors in the immediate aftermath of a disaster are strictly related to the presence or absence of attachment figures; when victims are with relatives or familiar people, they are more likely to behave within the group, relying on a leader. They decide to react actively but they are focused on helping and collaborating mostly with familiar people. People with strangers or alone instead tend to be more proactive and altruistic, helping people around. Results also underlined the importance of the environmental assessment in solidarity; the reaction of people is also influenced by the surrounding situation and the higher was the perception of physical danger, the more intense was the reaction and the solidarity showed.

This study also presents some limitations; this research was conducted in the immediate aftermath of the disaster; therefore, the chaotic situation made the data collection very challenging. However, the sample was not meant to be representative of the overall population as the non-probabilistic sampling methodology demonstrates; on the contrary, this work might serve as a pilot study for more systematic and scientific research in different cultural context and during different type of events**.**

## Conclusion

This paper should not be considered as an attempt to develop a new theory. On the contrary, it is an attempt to corroborate the already existing literature with empirical data that lack; affiliation approach and group approach can be useful to describe survivors’ behavior in the immediate aftermath of a disaster and it should be improved with specific disaster related factors, such as the danger perception or the environmental assessment. Furthermore, it might serve as a pilot study in advance of a more systematic and scientific research in different cultural context and during different type of events. Investigating how people react in the immediate aftermath of disasters, in fact, is essential to develop a more efficient and effective response strategy by disaster planners. The very first phase after the event is the most concerning one because usually authorities have little control over the situation due to complexity and uncertainty and actions or inactions, often uncontrolled and unmonitored, affect subsequent relief operations. Usually relief teams need time to arrive to the scene and people rely on themselves or on others in the proximity. The natural tendency to help others can be essential to reduce losses and to fill the temporal gap between the event and the arrival of the organized relief unit. This spontaneous behavior was characterized by collaboration and help: this is important in order to consider survivors not as a concern but as a resource.

## Corresponding Author

Dr. Andrea Bartolucci: andrea.bartolucci@anglia.ac.uk

## Data Availability

The data used can be accessed via https://dx.doi.org/10.6084/m9.figshare.4047561.v1

## Competing Interests

The authors have declared that no competing interests exist.
